# Influenza epidemiology and influenza vaccine effectiveness during the 2015–2016 season: results from the Global Influenza Hospital Surveillance Network

**DOI:** 10.1186/s12879-019-4017-0

**Published:** 2019-05-14

**Authors:** Joan Puig-Barberà, Ainara Mira-Iglesias, Elena Burtseva, Benjamin J. Cowling, Unal Serhat, Guillermo Miguel Ruiz-Palacios, Odile Launay, Jan Kyncl, Parvaiz Koul, Marilda M. Siqueira, Anna Sominina, on behalf of the Global Influenza Hospital Surveillance Network, A. Bagci Bosi, A. Goldstein, A. Mira-Iglesias, A. Sominina, B. Costa-Caetano, B. Cowling, B. Guglieri-López, C. El Guerche-Seblain, C. Mahé, C. Vlasich, E. Burtseva, F. Luzhao, F. Pradel, F. E. Moura, F. X. López-Labrador, G. M. Ruiz-Palacios, H. Schwarz-Chávarri, J. Díez-Domingo, J. Gurlichova, J. Kyncl, J. Mollar-Maseres, J. Puig-Barberà, K. Mubashir, K. Stolyarov, L. Kisteneva, L. Kolobukhina, M. Akcay Ciblak, M. Carballido-Fernández, M. de los Ángeles-Gutiérrez, M. Durusu Tanriover, M. Pisareva, M. Siqueira, M. Tortajada-Girbés, M. L. Guerrero-Almeida, O. Afanasieva, O. Launay, P. Cervantes, P. Koul, P. Loulergue, S. Raboni, S. Trushakova, S. Unal, V. Baselga-Moreno, V. Picot, W. Peng, Y. Qin, Y. Hongjie, Z. Lesieur, Z. Mandakova

**Affiliations:** 1 Fundación para el Fomento de la Investigación Sanitaria y Biomédica de la Comunidad Valenciana, FISABIO, Valencia, Spain; 2 Ivanovsky Institute of Virology FSBI “N.F, Gamaleya NRCEM” Ministry of Health, Moscow, Russian Federation; 3 School of Public Health, Li Ka Shing Faculty of Medicine, Hong Kong, Hong Kong, Special Administrative Region of China; 4 Turkish Society of Internal Medicine, Ankara, Turkey; 5 Salvador Zubirán National Institute of Medical Sciences and Nutrition (INCMNSZ), Vasco de Quiroga 15, Belisario Domínguez Sección 16, 14080 Tlalpan, CDMX Mexico; 6 INSERM, F-CRIN, Réseau National d’Investigation Clinique en Vaccinologie (I-REIVAC), CIC Cochin Pasteur, Paris, France and Université Paris Descartes, Sorbonne Paris Cité and Assistance Publique Hôpitaux de Paris, Hôpital Cochin, Paris, France; 7 National Institute of Public Health, Prague, Czech Republic; 8 Department of Internal and Pulmonary Medicine, Sher-i-Kashmir Institute of Medical Sciences (SKIMS), Soura, Bemina, Srinagar, Jammu & Kashmir 190011 India; 9 FIOCRUZ, Rio de Janeiro, Brazil; 10 Research Institute of Influenza, WHO National Influenza Centre of Russia and Ministry of Healthcare of the Russian Federation, St. Petersburg, Russian Federation

**Keywords:** Influenza, Virus, Surveillance, Vaccine, Hospitalization, Epidemiological study

## Abstract

**Background:**

The Global Influenza Hospital Surveillance Network is an international platform whose primary objective is to study severe cases of influenza requiring hospitalization.

**Methods:**

During the 2015–2016 influenza season, 11 sites in the Global Influenza Hospital Surveillance Network in nine countries (Russian Federation, Czech Republic, Turkey, France, China, Spain, Mexico, India, and Brazil) participated in a prospective, active-surveillance, hospital-based epidemiological study. Influenza infection was confirmed by reverse transcription-polymerase chain reaction. Influenza vaccine effectiveness (IVE) against laboratory-confirmed influenza was estimated using a test-negative approach.

**Results:**

9882 patients with laboratory results were included of which 2415 (24.4%) were positive for influenza, including 1415 (14.3%) for A(H1N1)pdm09, 235 (2.4%) for A(H3N2), 180 (1.8%) for A not subtyped, 45 (0.5%) for B/Yamagata-lineage, 532 (5.4%) for B/Victoria-lineage, and 33 (0.3%) for B not subtyped. Of included admissions, 39% were < 5 years of age and 67% had no underlying conditions. The odds of being admitted with influenza were higher among pregnant than non-pregnant women (odds ratio, 2.82 [95% confidence interval (CI), 1.90 to 4.19]). Adjusted IVE against influenza-related hospitalization was 16.3% (95% CI, 0.4 to 29.7). Among patients targeted for influenza vaccination, adjusted IVE against hospital admission with influenza was 16.2% (95% CI, − 3.6 to 32.2) overall, 23.0% (95% CI, − 3.3 to 42.6) against A(H1N1)pdm09, and − 25.6% (95% CI, − 86.3 to 15.4) against B/Victoria lineage.

**Conclusions:**

The 2015–2016 influenza season was dominated by A(H1N1)pdm09 and B/Victoria-lineage. Hospitalization with influenza often occurred in healthy and young individuals, and pregnant women were at increased risk of influenza-related hospitalization. Influenza vaccines provided low to moderate protection against hospitalization with influenza and no protection against the predominant circulating B lineage, highlighting the need for more effective and broader influenza vaccines.

**Electronic supplementary material:**

The online version of this article (10.1186/s12879-019-4017-0) contains supplementary material, which is available to authorized users.

## Background

Influenza surveillance is essential for tracking and controlling influenza infections and for assessing influenza vaccine effectiveness (IVE). Since 2012, the Global Influenza Hospital Surveillance Network (GIHSN) has run an annual prospective, active-surveillance, hospital-based study to collect epidemiological and virological data on influenza [1]. The aim of the GIHSN is to improve understanding of influenza epidemiology to better inform public health policy decisions.

During the 2015–2016 influenza season, the GIHSN included 11 coordinating sites and 27 hospitals in nine countries (St. Petersburg and Moscow, Russian Federation; Prague, Czech Republic; Ankara, Turkey; Paris, France; Beijing, China; Valencia, Spain; Tlalpan, Mexico; Jammu and Kashmir, India; and Fortaleza and Curitiba, Brazil). All sites in the GIHSN share a common core protocol, follow standard operating procedures, use a shared questionnaire to collect patient information, and perform reverse transcription-polymerase chain reaction to confirm influenza infection [1]. Thus, the GIHSN can attain large sample sizes and relevant data on severe influenza and IVE among hospitalized individuals from geographically disperse regions. In addition, several limitations of other surveillance systems are avoided or adjusted for, such as non-systematic sampling and incomplete case ascertainment, as well as a lack of comparison groups, adjustment for confounders, and consensus about case definitions [2–4]. Results have been published for the GIHSN’s first three seasons (2012–2013 [5, 6], 2013–2014 [7], and 2014–2015 [8]). Here, we present the influenza epidemiology and IVE results by age and influenza strain for the 2015–2016 influenza season.

## Methods

### Study design

The GIHSN was initiated by Sanofi Pasteur in 2011 to fill the gap in influenza epidemiology and public health knowledge. The GIHSN is a public-private partnership between Sanofi Pasteur and several institutions that are affiliated with national health authorities (including the WHO National Influenza Centers, national ministries of health, and China’s Centers for Diseases Control and Prevention). Each of these institutions acts as a coordinating site and supervises a local network of hospitals. Not-for-profit institutions with proposals aligned with the GIHSN scope and study design are eligible to apply for grants from the Foundation for Influenza Epidemiology. Sanofi Pasteur participated in the design of the study but did not participate in the collection, management, or analysis of data.

The methodology for the GIHSN study has previously been described in detail [1, 5, 6]. Briefly, the study included patients who had been admitted to one of the participating hospitals for acute illness possibly related to influenza within the last 48 h. The patients had to be residents in the predefined hospital’s catchment area for at least 6 months, not institutionalized, and not discharged from a hospital within 30 days of the current admission. Onset of symptoms had to be within 7 days prior to admission. Acute illness in patients aged ≥5 years had to meet the European Centre for Disease Prevention and Control clinical case definition of influenza-like illness (ILI) [9]; include one of the following general symptoms: fever or feverishness, malaise, myalgia or headache; and include one of the following respiratory complaints: shortness of breath, sore throat, or cough. Patients aged < 5 years were recruited if they presented with any of the signs and symptoms described in Additional file 1: Table S1. Patient eligibility was assessed by research staff using admission rolls, clinical records, and information obtained from the patient after consent. Each site defined the sample collection period according to previous experience in local influenza epidemics (see Additional file 2: Table S2). For each patient, a common standardized questionnaire was completed by face-to-face interview or by searching clinical records. Collected information included age, sex, number of chronic underlying conditions, previous admissions to hospital in the last 12 months, number of visits to a general practitioner in the last 3 months, smoking habits, socioeconomic class (according to occupation), days from onset of symptoms to swabbing, and epidemiological week at admission. The influenza vaccination status of each patient was also collected by face-to face interview, patient records, clinical records, or registries, including the name of the vaccine received and the date of vaccination. Two respiratory swabs were taken from each patient (nasal and nasopharyngeal swabs from patients < 14 years, pharyngeal and nasopharyngeal swabs from patients ≥14 years) and combined to detect the presence of influenza A (H1N1pdm09 and H3N2 subtypes) and B (Yamagata and Victoria lineages) by real-time reverse-transcription polymerase chain reaction. All included patients, or their parents or legal guardians, provided written informed consent.

### Statistical analysis

Statistical analyses were performed using Stata version 14.2 (College Station, TX, USA). Differences between categories were estimated by the Pearson Chi-square or Fisher exact test as appropriate. When comparing nested models, *P*-values for interactions were obtained by likelihood ratio test. *P*-values below 0.05 were considered statistically significant. Conditional plots were used to describe complex relationships between age, chronic conditions and influenza infection [10].

IVE was estimated using the test-negative approach as (1 − odds ratio [OR]) × 100, where the OR was calculated by mixed effects logistic regression comparing the vaccine coverage rates between influenza-positive and influenza-negative cases, after adjusting for potential confounders. Appropriate variables were included depending on the model. For the IVE model, age was divided into deciles and modelled using restricted cubic splines; sex was a categorical variable; social class was a categorical variable with four levels (qualified, skilled, low or unskilled, and unknown); number of comorbidities was a categorical variable with three levels (none, one, more than one); vaccination status had two levels (yes/no); time from onset to swab was a categorical variable with three levels (0 to 4, 5 to 7, 8 to 9); epidemiological week at admission was modelled using restricted cubic splines. The number of knots for age and epidemiological week was chosen using the Akaike information criterion [11]. IVE was not estimated for individual influenza vaccines. Heterogeneity between sites was controlled by including the site as a random effect in the models. All included patients were considered in the descriptive analysis, but records with missing values for outcome, exposure, or with potential confounders, and individuals with contraindications for vaccination or with previous influenza infections, were excluded from the IVE analysis. IVE values were considered heterogeneous if the I^2^ statistic was > 50%.

## Results

### Patients included in the epidemiological analysis and identified viruses

A total of 18,360 eligible admissions were identified by the 11 coordinating sites during the 2015–2016 influenza season. Of these, 9882 admissions (53.8%) met the inclusion criteria and were included in the study (Table 1). The main reasons for exclusion were the absence of ILI symptoms for subjects ≥5 years of age (*n* = 3886, 21.2%) and recruitment during weeks without influenza circulation (*n* = 1524, 8.3%). No cases at the Fortaleza site met the inclusion criteria.

**Table 1:** Exclusions, inclusions, and RT-PCR results for included admissions

Category	n (%)											
	St. Petersburg	Moscow	Czech Republic	Turkey	France	Beijing	Valencia	Mexico	India	Fortaleza	Curitiba	Total
	*N* = 2247	*N* = 2457	*N* = 248	*N* = 1351	*N* = 169	*N* = 2947	*N* = 6795	*N* = 1034	*N* = 371	*N* = 157	*N* = 584	N = 18,360
Excluded from the analysis												
Non-resident	10 (0.4)	84 (3.4)	1 (0.4)	10 (0.7)	0 (0.0)	1 (< 0.1)	25 (0.4)	249 (24.1)	5 (1.3)	3 (1.9)	0 (0.0)	388 (2.1)
Institutionalized	0 (0.0)	32 (1.3)	6 (2.4)	25 (1.9)	0 (0.0)	2 (0.1)	0 (0.0)	5 (0.5)	0 (0.0)	1 (0.6)	3 (0.5)	74 (0.4)
Unable to communicate	20 (0.9)	112 (4.6)	14 (5.6)	96 (7.1)	0 (0.0)	0 (0.0)	59 (0.9)	56 (5.4)	0 (0.0)	0 (0.0)	65 (11.1)	422 (2.3)
Did not provide consent	80 (3.6)	26 (1.1)	21 (8.5)	11 (0.8)	0 (0.0)	0 (0.0)	16 (0.2)	40 (3.9)	1 (0.3)	0 (0.0)	3 (0.5)	198 (1.1)
Previous discharge from hospital < 30 d	11 (0.5)	80 (3.3)	6 (2.4)	266 (19.7)	25 (14.8)	24 (0.8)	66 (1.0)	208 (20.1)	9 (2.4)	47 (29.9)	89 (15.2)	831 (4.5)
No ILI symptoms, ≥ 5 y of age^a^	0 (0.0)	34 (1.4)	60 (24.2)	3 (0.2)	2 (1.2)	0 (0.0)	3708 (54.6)	60 (5.8)	0 (0.0)	19 (12.1)	0 (0.0)	3886 (21.2)
Swabbed > 7 d after onset of symptoms (all ages)^b^	55 (2.4)	134 (5.5)	16 (6.5)	72 (5.3)	0 (0.0)	28 (1.0)	226 (3.3)	237 (22.9)	17 (4.6)	18 (11.5)	218 (37.3)	1021 (5.6)
Sample inadequate^c^	0 (0.0)	0 (0.0)	0 (0.0)	0 (0.0)	0 (0.0)	0 (0.0)	2 (< 0.1)	0 (0.0)	0 (0.0)	0 (0.0)	0 (0.0)	2 (< 0.1)
Previous influenza infection^d^	3 (0.1)	1 (< 0.1)	1 (0.4)	2 (0.1)	0 (0.0)	0 (0.0)	2 (< 0.1)	0 (0.0)	2 (0.5)	0 (0.0)	0 (0.0)	11 (0.1)
Recruited in weeks without laboratory-confirmed influenza cases	0 (0.0)	0 (0.0)	0 (0.0)	57 (4.2)	1 (0.6)	735 (24.9)	547 (8.1)	83 (8.0)	7 (1.9)	69 (43.9)	25 (4.3)	1524 (8.3)
Included in the analysis	2071 (92.2)	1934 (78.7)	124 (50.0)	704 (52.1)	141 (83.4)	2157 (73.2)	2145 (31.6)	96 (9.3)	331 (89.2)	0 (0.0)	179 (30.7)	9882 (53.8)
RT-PCR result												
Negative for influenza	1331 (64.3)	1256 (64.9)	82 (66.1)	561 (79.7)	97 (68.8)	1804 (83.6)	1828 (85.2)	51 (53.1)	294 (88.8)	0 (0.0)	163 (27.9)	7467 (75.6)
Positive for influenza	740 (35.7)	678 (35.1)	42 (33.9)	143 (20.3)	44 (31.2)	353 (16.4)	317 (14.8)	45 (46.9)	37 (11.2)	0 (0.0)	16 (2.7)	2415 (24.4)
Influenza A(H1N1)pdm09	527 (25.4)	462 (23.9)	23 (18.5)	68 (9.7)	25 (17.7)	108 (5.0)	163 (7.6)	21 (21.9)	5 (1.5)	0 (0.0)	13 (2.2)	1415 (14.3)
Influenza A(H3N2)	16 (0.8)	44 (2.3)	2 (1.6)	56 (8.0)	1 (0.7)	82 (3.8)	3 (0.1)	13 (13.5)	18 (5.4)	0 (0.0)	0 (0.0)	235 (2.4)
Influenza A/not subtyped	104 (5.0)	11 (0.6)	1 (0.8)	10 (1.4)	0 (0.0)	0 (0.0)	50 (2.3)	4 (4.2)	0 (0.0)	0 (0.0)	0 (0.0)	180 (1.8)
Influenza B/Yamagata	2 (0.1)	1 (0.1)	8 (6.5)	11 (1.6)	0 (0.0)	12 (0.6)	0 (0.0)	2 (2.1)	9 (2.7)	0 (0.0)	0 (0.0)	45 (0.5)
Influenza B/Victoria	96 (4.6)	153 (7.9)	8 (6.5)	0 (0.0)	16 (11.3)	155 (7.2)	92 (4.3)	4 (4.2)	5 (1.5)	0 (0.0)	3 (0.5)	532 (5.4)
Influenza B/not subtyped	8 (0.4)	7 (0.4)	1 (0.8)	1 (0.1)	2 (1.4)	0 (0.0)	12 (0.6)	2 (2.1)	0 (0.0)	0 (0.0)	0 (0.0)	33 (0.3)

*Abbreviations*: *ILI* influenza-like illness, *RT-PCR* reverse transcriptase-polymerase chain reaction

^a^9 missing for Turkey

^b^98 missing for Turkey

^c^21 missing for Moscow, 1 for India, and 2 for Curitiba

^d^21 missing for Moscow

Approximately 24% of the included patients were positive for influenza. The most common strain detected was A(H1N1)pdm09 (58.6% of influenza positives), followed by B/Victoria-lineage (22.0%) and A(H3N2) (9.7%). Approximately 9% of influenza-positive samples could not be subtyped.

### Description of the 2015–2016 influenza season across sites

**Fig. 1:**
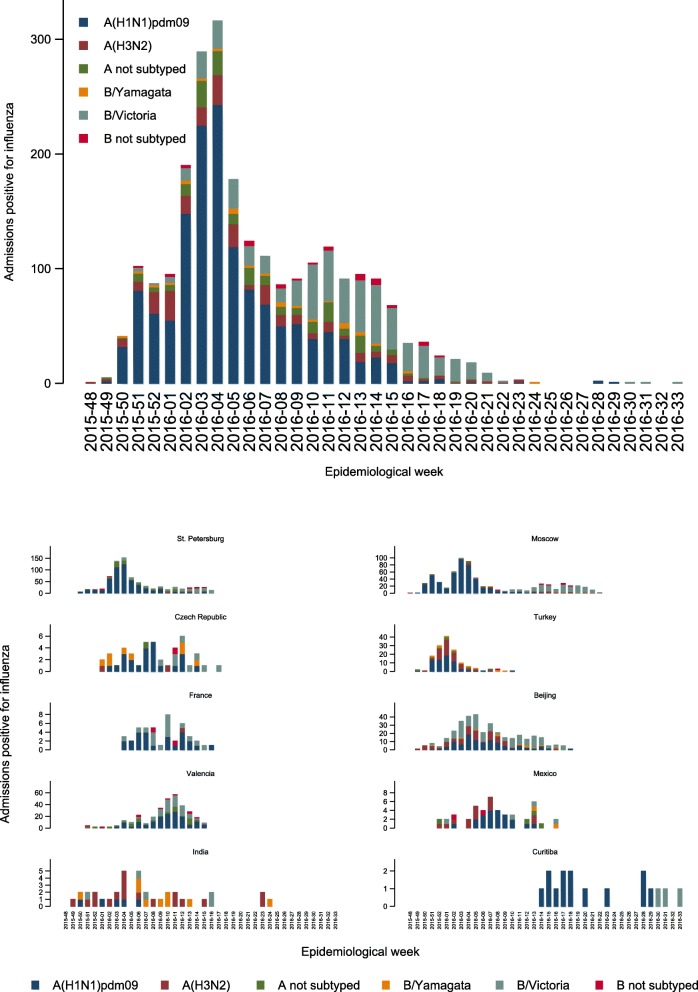
Admissions with influenza by epidemiological week and virus type, subtype, or lineage overall and by site

Influenza infections were detected over 38 weeks, with the peak at week 4 of 2016 (Fig. 1). The earliest start of the influenza season was in Moscow (week 48 of 2015), where influenza-positive admissions occurred over a span of 27 weeks in two waves, the first due to A(H1N1)pdm09 and the second due to B/Victoria-lineage. The latest influenza-positive admission (week 33 of 2016) was in Curitiba, Brazil, although few cases of confirmed influenza were detected at that site.

The proportion of samples positive for influenza differed between sites from 2.7% in Curitiba to 46.9% in Mexico (Table 1; *P* <  0.0001 by test of homogeneity, equal odds). A(H1N1)pdm09 was the most frequently detected influenza virus in St. Petersburg (71.2% of positives), Moscow (68.1%), Czech Republic (54.8%), Turkey (47.6%), France (56.8%), Valencia (51.4%), Mexico (46.7%), and Curitiba (81.3%) (Table 1). B/Victoria-lineage was the second-most frequently detected influenza virus in Moscow (22.6%), St. Petersburg (13.0%), France (36.4%), Valencia (29.0%) and Curitiba (18.8%) and was the most common influenza virus in Beijing (43.9%). Influenza A(H3N2) was the predominant strain in India (48.6%) (Fig. 1 and Table 1).

### Main characteristics of included patients

Of the 9882 included admissions, 39.0% were < 5 years of age, 38.0% were 5–64 years of age, and 23.0% were ≥ 65 years of age (Table 2). Just over half of the included patients were male (*n* = 5380; 54.4%) and more than half (615/1051; 58.5%) of the admitted women 15–45 years of age were pregnant (6.2% of all included patients).

**Table 2:** Characteristics of included admissions overall and by site

Category	n (%)										
	St. Petersburg	Moscow	Czech Republic	Turkey	France	Beijing	Valencia	Mexico	India	Curitiba	Total
	*N* = 2071	*N* = 1934	*N* = 124	*N* = 704	*N* = 141	*N* = 2157	*N* = 2145	*N* = 96	*N* = 331	*N* = 179	*N* = 9882
Age (y), median [IQR]	2 [0–12]	20 [3–29]	48 [31–69]	6 [0–65]	66 [51–79]	9 [3–66]	70 [39–82]	42 [20–58]	37 [0–65]	1 [0–6]	20 [2–61]
Age group^a^											
0–1 y	813 (39.3)	239 (12.4)	0 (0.0)	247 (35.1)	0 (0.0)	309 (14.3)	317 (14.8)	7 (7.3)	111 (33.5)	102 (57.0)	2145 (21.7)
2–4 y	550 (26.6)	365 (18.9)	0 (0.0)	96 (13.7)	0 (0.0)	572 (26.5)	82 (3.8)	6 (6.3)	13 (3.9)	27 (15.1)	1711 (17.3)
5–17 y	261 (12.6)	228 (11.8)	0 (0.0)	69 (9.8)	0 (0.0)	258 (12.0)	53 (2.5)	10 (10.4)	6 (1.8)	23 (12.8)	908 (9.2)
18–49 y	356 (17.2)	1000 (51.7)	66 (53.2)	41 (5.8)	32 (22.7)	216 (10.0)	190 (8.9)	33 (34.4)	59 (17.8)	8 (4.5)	2001 (20.2)
50–64 y	69 (3.3)	67 (3.5)	18 (14.5)	70 (10.0)	32 (22.7)	239 (11.1)	275 (12.8)	23 (24.0)	41 (12.4)	13 (7.3)	847 (8.6)
65–74 y	6 (0.3)	15 (0.8)	16 (12.9)	59 (8.4)	27 (19.1)	244 (11.3)	352 (16.4)	4 (4.2)	64 (19.3)	5 (2.8)	792 (8.0)
75–84 y	15 (0.7)	17 (0.9)	18 (14.5)	85 (12.1)	29 (20.6)	260 (12.1)	489 (22.8)	11 (11.5)	31 (9.4)	1 (0.6)	956 (9.7)
≥ 85 y	1 (< 0.1)	3 (0.2)	6 (4.8)	36 (5.1)	21 (14.9)	59 (2.7)	387 (18.0)	2 (2.1)	6 (1.8)	0 (0.0)	521 (5.3)
Sex											
Male	1180 (57.0)	1002 (51.8)	67 (54.0)	378 (53.7)	63 (44.7)	1223 (56.7)	1151 (53.7)	42 (43.8)	180 (54.4)	94 (52.5)	5380 (54.4)
Female	891 (43.0)	932 (48.2)	57 (46.0)	326 (46.3)	78 (55.3)	934 (43.3)	994 (46.3)	54 (56.3)	151 (45.6)	85 (47.5)	4502 (45.6)
Chronic conditions											
0	1872 (90.4)	1718 (88.8)	60 (48.4)	337 (47.9)	42 (29.8)	1529 (70.9)	742 (34.6)	32 (33.3)	156 (47.1)	118 (65.9)	6606 (66.8)
1	164 (7.9)	172 (8.9)	37 (29.8)	177 (25.1)	47 (33.3)	406 (18.8)	611 (28.5)	29 (30.2)	84 (25.4)	40 (22.3)	1767 (17.9)
> 1	35 (1.7)	44 (2.3)	27 (21.8)	190 (27.0)	52 (36.9)	222 (10.3)	792 (36.9)	35 (36.5)	91 (27.5)	21 (11.7)	1509 (15.3)
Hospitalized within ≤12 mo											
No	1408 (68.0)	1577 (81.5)	99 (79.8)	385 (54.7)	76 (53.9)	1774 (82.2)	1448 (67.5)	62 (64.6)	202 (61.0)	137 (76.5)	7168 (72.5)
Yes	663 (32.0)	357 (18.5)	25 (20.2)	319 (45.3)	65 (46.1)	383 (17.8)	697 (32.5)	34 (35.4)	129 (39.0)	42 (23.5)	2714 (27.5)
Underlying conditions											
Cardiovascular disease	59 (2.8)	100 (5.2)	38 (30.6)	219 (31.1)	60 (42.6)	427 (19.8)	842 (39.3)	30 (31.3)	94 (28.4)	19 (10.6)	1888 (19.1)
Chronic obstructive pulmonary disease	21 (1.0)	18 (0.9)	4 (3.2)	98 (13.9)	30 (21.3)	252 (11.7)	510 (23.8)	7 (7.3)	85 (25.7)	14 (7.8)	1039 (10.5)
Asthma	44 (2.1)	19 (1.0)	10 (8.1)	60 (8.5)	8 (5.7)	52 (2.4)	170 (7.9)	14 (14.6)	18 (5.4)	29 (16.2)	424 (4.3)
Immunodeficiency/organ transplant	11 (0.5)	0 (0.0)	6 (4.8)	33 (4.7)	8 (5.7)	1 (< 0.1)	19 (0.9)	10 (10.4)	3 (0.9)	1 (0.6)	92 (0.9)
Diabetes	14 (0.7)	19 (1.0)	16 (12.9)	88 (12.5)	28 (19.9)	98 (4.5)	515 (24.0)	12 (12.5)	37 (11.2)	13 (7.3)	840 (8.5)
Chronic renal impairment	14 (0.7)	54 (2.8)	6 (4.8)	54 (7.7)	22 (15.6)	15 (0.7)	247 (11.5)	16 (16.7)	26 (7.9)	0 (0.0)	454 (4.6)
Chronic neuromuscular disease	47 (2.3)	19 (1.0)	3 (2.4)	59 (8.4)	4 (2.8)	22 (1.0)	38 (1.8)	7 (7.3)	5 (1.5)	8 (4.5)	212 (2.1)
Active neoplasm	6 (0.3)	8 (0.4)	12 (9.7)	57 (8.1)	18 (12.8)	28 (1.3)	148 (6.9)	4 (4.2)	22 (6.6)	1 (0.6)	304 (3.1)
Chronic liver disease	15 (0.7)	21 (1.1)	3 (2.4)	8 (1.1)	4 (2.8)	9 (0.4)	73 (3.4)	3 (3.1)	1 (0.3)	0 (0.0)	137 (1.4)
Autoimmune disease	13 (0.6)	15 (0.8)	4 (3.2)	10 (1.4)	0 (0.0)	8 (0.4)	27 (1.3)	11 (11.5)	14 (4.2)	2 (1.1)	104 (1.1)
Pregnant (women 15–45 y)	0 (0.0)	596 (30.8)	2 (1.6)	2 (0.3)	4 (2.8)	0 (0.0)	3 (0.1)	0 (0.0)	4 (1.2)	4 (2.2)	615 (6.2)
Obese^b,c^	202 (9.8)	200 (10.3)	36 (29.0)	116 (19.5)	23 (16.3)	238 (11.0)	529 (24.7)	25 (26.3)	31 (9.4)	25 (14.0)	1425 (14.4)
Outpatient consultations within ≤3 mo^d^											
0	927 (44.8)	655 (33.9)	43 (34.7)	164 (23.3)	17 (12.1)	1039 (48.2)	214 (10.0)	38 (39.6)	46 (13.9)	47 (26.3)	2151 (21.8)
1	660 (31.9)	447 (23.1)	34 (27.4)	186 (26.4)	54 (38.6)	1022 (47.4)	246 (11.5)	18 (18.8)	54 (16.3)	74 (41.3)	2812 (28.5)
≥ 2	484 (23.4)	832 (43.0)	47 (37.9)	354 (50.3)	69 (49.3)	96 (4.45)	1685 (78.6)	40 (41.7)	231 (69.8)	58 (32.4)	4918 (49.8)
Smoking habits (≥ 18 y)^e^											
Never smoked	221 (49.4)	571 (51.8)	70 (56.5)	153 (58.0)	64 (45.4)	570 (56.0)	749 (44.2)	39 (53.4)	103 (51.2)	8 (29.6)	2548 (50.1)
Past smoker	57 (12.8)	249 (22.6)	15 (12.1)	87 (33.0)	41 (29.1)	270 (26.5)	569 (33.6)	29 (39.7)	74 (36.8)	13 (48.1)	1404 (27.6)
Current smoker	169 (37.8)	282 (25.6)	39 (31.5)	24 (9.1)	36 (25.5)	178 (17.5)	375 (22.2)	5 (6.8)	24 (11.9)	6 (22.2)	1138 (22.4)
Functional impairment status (Barthel index, patients ≥65 y)^f^											
Total (0–15)	0 (0.0)	0 (0.0)	1 (2.5)	21 (11.7)	0 (0.0)	37 (11.8)	116 (9.5)	0 (0.0)	5 (5.1)	0 (0.0)	180 (8.9)
Severe (20–35)	0 (0.0)	0 (0.0)	0 (0.0)	12 (6.7)	1 (1.3)	10 (3.2)	45 (3.7)	1 (5.9)	8 (8.2)	0 (0.0)	77 (3.8)
Moderate (40–55)	0 (0.0)	0 (0.0)	1 (2.5)	14 (7.8)	4 (5.2)	38 (12.1)	72 (5.9)	2 (11.8)	23 (23.5)	0 (0.0)	154 (7.7)
Mild (60–90)	4 (18.2)	7 (20.0)	5 (12.5)	71 (39.7)	16 (20.8)	172 (55.0)	230 (18.7)	8 (47.1)	41 (41.8)	0 (0.0)	554 (27.5)
Minimal (95–100)	18 (81.8)	28 (80.0)	33 (82.5)	61 (34.1)	56 (72.7)	56 (17.9)	764 (62.3)	6 (35.3)	21 (21.4)	5 (100.0)	1048 (52.1)
Time from onset of symptoms to swabbing											
0–2 d	1102 (53.2)	1025 (53.1)	41 (33.1)	133 (19.1)	63 (44.7)	503 (23.3)	446 (20.8)	17 (17.9)	34 (10.3)	37 (20.7)	3401 (34.5)
3–4 d	656 (31.7)	619 (32.1)	46 (37.1)	251 (36.1)	41 (29.1)	628 (29.1)	820 (38.3)	22 (23.2)	130 (39.3)	98 (54.7)	3311 (33.6)
5–7 d	313 (15.1)	281 (14.6)	36 (29.0)	301 (43.2)	37 (26.2)	899 (41.7)	681 (31.8)	48 (50.5)	164 (49.5)	43 (24.0)	2803 (28.4)
8–9 d	0 (0.0)	4 (0.2)	1 (0.8)	11 (1.6)	0 (0.0)	126 (5.8)	196 (9.1)	8 (8.4)	3 (0.9)	1 (0.6)	350 (3.5)
> 10 or missing	–	5	–	8	–	1	2	1	–	–	17
Vaccinated for influenza during 2015–2016^g^	45 (2.2)	87 (4.5)	7 (5.6)	49 (7.0)	60 (42.6)	262 (12.1)	923 (43.0)	19 (19.8)	15 (4.5)	58 (32.4)	1525 (15.4)
≥ 14 d from ILI onset^h^	45 (100.0)	87 (100.0)	7 (100.0)	41 (83.7)	60 (100.0)	260 (99.2)	912 (98.8)	5 (26.3)	15 (100.0)	46 (79.3)	1478 (96.9)

*Abbreviations*: *ILI* influenza-like illness

^a^1 missing for Turkey

^b^29 missing for Turkey

^c^Determined from the body mass index according to age and sex following the World Health Organization guidelines [12]

^d^1 missing for France; Beijing categories were 0, 1–3, and ≥ 4

^e^28 missing in Turkey

^f^2 missing for Turkey, 250 for Beijing, 1 for Valencia, 3 for India, 1 for Curitiba

^g^2 missing for St. Petersburg

^h^Percentages are calculated relative to vaccinated patients

Approximately one-third (*n* = 3276; 33.2%) of the admissions had chronic conditions, most of which were cardiovascular disease (*n* = 1888; 19.1%), chronic obstructive pulmonary disease (*n* = 1039; 10.5%), diabetes (*n* = 840; 8.5%), renal impairment (*n* = 454; 4.6%), and asthma (*n* = 424; 4.3%). Immunodeficiency, neuromuscular disease, active neoplasm, liver disease, and autoimmune disease accounted for < 4% of patients with chronic conditions.

Most patients (*n* = 7168; 72.5%) had not been hospitalized in the 12 months before the current admission. Half of the hospitalized adults (≥ 18 years old) had never smoked (*n* = 2548; 50.1%), 27.6% (*n* = 1404) were past smokers, and 22.4% (*n* = 1138) were current smokers. According to World Health Organization criteria [12], 14.4% (*n* = 1425) of patients were obese. Approximately 20% of older adults (≥ 65 years of age) had a functional impairment status between moderate and total on the Barthel Index.

Swabs were obtained within 4 days after the onset of symptoms in 67.9% of patients (*n* = 6712). The seasonal influenza vaccine had been administered to 1525 patients (15.4%), most of whom (96.9%) had been vaccinated at least 14 days before the onset of ILI symptoms.

### Characteristics of included patients across sites

Proportions of younger patients were highest in Curitiba (median age = 1 years) and St. Petersburg (median age = 2 years) (Table 2). Children < 5 years of age accounted for 72.1% of included admissions in Curitiba and 65.8% in St. Petersburg. The Czech Republic and France did not recruit subjects < 18 years of age. Patients were mostly young adults (18–49 years of age) in the Czech Republic (53.2%) and Moscow (51.7%). By contrast, in Valencia (57.2%) and France (54.6%), most included admissions were ≥ 65 years of age.

Admissions were more frequently males than females at all sites (51.8 to 57.0%) except France (44.7%) and Mexico (43.8%). Proportions of patients without comorbidities were highest in St. Petersburg (90.4%) and Moscow (88.8%), followed by Beijing (70.9%), Curitiba (65.9%), Czech Republic (48.4%), Turkey (47.9%), India (47.1%), Valencia (34.6%), Mexico (33.3%), and France (29.8%). Cardiovascular disease was the most common chronic condition at all sites except Curitiba, where asthma was the most common (Table 2).

In Moscow, 30.8% of the included patients were pregnant women. By contrast, at the rest of the sites, pregnant women accounted for < 3% of the included patients.

The proportion of obese patients was highest in the Czech Republic (*n* = 36; 29.0%), Mexico (*n* = 25; 26.3%), and Valencia (*n* = 529; 24.7%) and lowest in India (*n* = 31; 9.4%), St. Petersburg (*n* = 202; 9.8%), and Moscow (*n* = 200; 10.3%).

Visits to general practitioners during the 3 months before hospitalization were the least common in Beijing (*n* = 1118; 51.8%), St. Petersburg (1144; 55.3%), and Mexico (*n* = 58; 60.5%) and the most common in Valencia (*n* = 1931; 90.0%), France (*n* = 123; 87.9%) and India (*n* = 285; 86.1%).

The proportion of adult patients who had never smoked ranged from 29.6% (*n* = 8) in Curitiba to 58.0% (*n* = 153) in Turkey. The proportion of adult patients that currently smoked ranged from 6.8% (n = 5) in Mexico to 37.8% (*n* = 169) in St. Petersburg.

Moderate to total functional impairment in older adults (≥ 65 years of age) was least common in St. Petersburg, Moscow, and Curitiba (0.0%) and highest in India (36.7%). Vaccination coverage varied substantially between sites (Table 2). Vaccination coverage rates were (in decreasing order) 43.0% (*n* = 923) in Valencia, 42.6% (*n* = 60) in France, 32.4% (n = 58) in Curitiba, 19.8% (n = 19) in Mexico, 12.1% (*n* = 262) in Beijing, 7.0% (*n* = 49) in Turkey, 5.6% (*n* = 7) in the Czech Republic, 4.5% in India (n = 15) and Moscow (*n* = 87), and 2.2% (*n* = 45) in St. Petersburg. Among vaccinated individuals, the vaccine had been administered ≥14 days before the onset of symptoms to 26.3% (n = 5) of admissions in Mexico, 79.3% (*n* = 46) in Curitiba, 83.7% (*n* = 41) in Turkey, and nearly all admissions (99 to 100%) at other sites. From the information available, 87% of vaccinated individuals had received a trivalent inactivated influenza vaccine (data not shown). Almost all of the remaining 13% also received a trivalent vaccine, based on the vaccines available at each study site (Additional file 3: Table S3).

### Admission with influenza according to age and variability by influenza virus

**Fig. 2:**
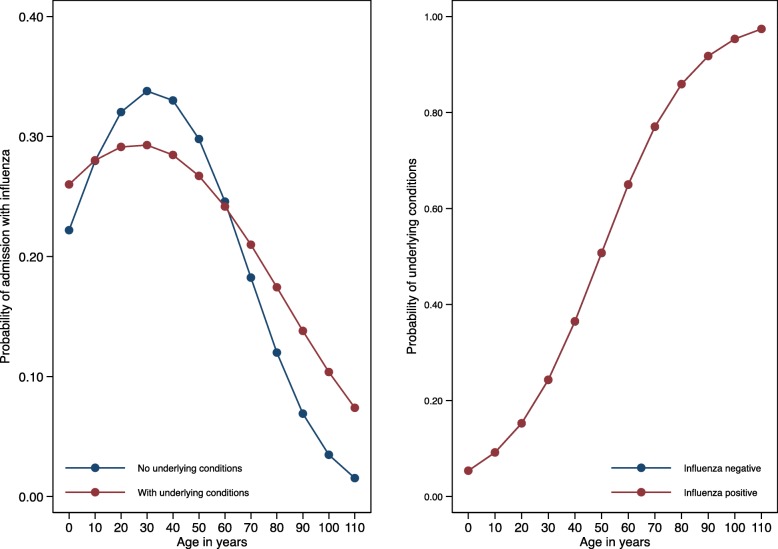
Admissions with influenza by age and underlying conditions. Adjusted by the interaction of age with chronic conditions, influenza immunization, and site clustering effects

Influenza positivity appeared to be related to age. Admissions positive for influenza were younger than admissions negative for influenza, regardless of having underlying conditions (Fig. 2). More than three-quarters (76.1%) of influenza-positive admissions were < 50 years of age (Table 3, Fig. 2). Admissions positive for A(H1N1)pdm09 were generally younger than admissions negative for influenza, positive for A(H3N2), or positive for B/Yamagata-lineage but older than admissions positive for B/Victoria-lineage (Table 3).

**Table 3:** Characteristics of included admissions according to RT-PCR result

Characteristic	n (%)							
	Influenza negative	Any influenza	A(H1N1)pdm09	A(H3N2)	A not subtyped	B/Yamagata	B/Victoria	B not subtyped
	*N* = 7467	*N* = 2415	*N* = 1415	*N* = 235	*N* = 180	*N* = 45	*N* = 532	N = 33
Age (y), median (IQR)	20 (2–65)	18 (3–46)	19 (2–43)	30 (3–71)	4 (1–42)	37 (4–57)	10 (4–31)	25 (5–56)
P vs. influenza negative	–	0.136	0.794	< 0.001	0.001	0.231	< 0.001	0.595
Age group^a^								
< 1 y	1138 (15.2)	232 (9.6)	135 (9.5)	16 (6.8)	45 (25.0)	7 (15.6)	32 (6.0)	4 (12.1)
1–4 y	1823 (24.4)	663 (27.5)	423 (29.9)	49 (20.9)	47 (26.1)	5 (11.1)	141 (26.5)	3 (9.1)
5–17 y	613 (8.2)	295 (12.2)	135 (9.5)	12 (5.1)	15 (8.3)	6 (13.3)	123 (23.1)	8 (24.2)
18–49 y	1352 (18.1)	649 (26.9)	409 (28.9)	57 (24.3)	33 (18.3)	10 (22.2)	134 (25.2)	7 (21.2)
50–64 y	645 (8.6)	202 (8.4)	137 (9.7)	25 (10.6)	13 (7.2)	9 (20.0)	17 (3.2)	4 (12.1)
65–74 y	640 (8.6)	152 (6.3)	71 (5.0)	27 (11.5)	9 (5.0)	5 (11.1)	38 (7.1)	4 (12.1)
75–84 y	796 (10.7)	160 (6.6)	76 (5.4)	36 (15.3)	12 (6.7)	3 (6.7)	35 (6.6)	1 (3.0)
≥ 85 y	459 (6.1)	62 (2.6)	29 (2.0)	13 (5.5)	6 (3.3)	0 (0.0)	12 (2.3)	2 (6.1)
*P* vs. influenza negative	–	< 0.001	< 0.001	< 0.001	0.008	0.03	< 0.001	0.010
Sex								
Male	4112 (55.1)	1268 (52.5)	738 (52.2)	125 (53.2)	101 (56.1)	24 (53.3)	278 (52.3)	19 (57.6)
Female	3355 (44.9)	1147 (47.5)	677 (47.8)	110 (46.8)	79 (43.9)	21 (46.7)	254 (47.7)	14 (42.4)
*P* vs. influenza negative	–	0.028	0.041	0.569	0.832	0.815	0.208	0.893
Underlying chronic conditions								
No	4869 (65.2)	1737 (71.9)	1038 (73.4)	132 (56.2)	125 (69.4)	22 (48.9)	414 (77.8)	20 (60.6)
Yes	2598 (34.8)	678 (28.1)	377 (26.6)	103 (43.8)	55 (30.6)	23 (51.1)	118 (22.2)	13 (39.4)
*P* vs. influenza negative	–	< 0.001	< 0.001	0.004	0.259	0.022	< 0.001	0.490
Hospitalized within ≤12 mo								
No	5390 (72.2)	1778 (73.6)	1066 (75.3)	160 (68.1)	113 (62.8)	33 (73.3)	405 (76.1)	19 (57.6)
Yes	2077 (27.8)	637 (26.4)	349 (24.7)	75 (31.9)	67 (37.2)	12 (26.7)	127 (23.9)	14 (42.4)
*P* vs. influenza negative	–	0.168	0.015	0.168	0.005	0.864	0.049	0.107
Underlying chronic conditions								
Cardiovascular disease	1543 (20.7)	345 (14.3)	188 (13.3)	57 (24.3)	27 (15.0)	12 (26.7)	59 (11.1)	6 (18.2)
*P* vs. influenza negative	–	< 0.001	< 0.001	0.182	0.068	0.322	< 0.001	0.79
Chronic obstructive pulmonary disease	860 (11.5)	179 (7.4)	88 (6.2)	37 (15.7)	13 (7.2)	5 (11.1)	35 (6.6)	5 (15.2)
*P* vs. influenza negative	–	< 0.001	< 0.001	0.047	0.077	1.0	< 0.001	0.409
Asthma	339 (4.5)	85 (3.5)	46 (3.3)	12 (5.1)	13 (7.2)	2 (4.4)	12 (2.3)	2 (6.1)
*P* vs. influenza negative	–	0.031	0.029	0.682	0.086	1.0	0.013	0.656
Immunodeficiency/organ transplant	66 (0.9)	26 (1.1)	14 (1.0)	6 (2.6)	2 (1.1)	1 (2.2)	2 (0.4)	1 (3.0)
*P* vs. influenza negative	–	0.391	0.698	0.009	0.674	0.333	0.325	0.250
Diabetes	704 (9.4)	136 (5.6)	72 (5.1)	21 (8.9)	12 (6.7)	7 (15.6)	23 (4.3)	4 (12.1)
*P* vs. influenza negative	–	< 0.001	< 0.001	0.799	0.216	0.162	< 0.001	0.538
Chronic renal impairment	336 (4.5)	118 (4.9)	63 (4.5)	17 (7.2)	12 (6.7)	3 (6.7)	22 (4.1)	4 (12.1)
*P* vs. influenza negative	–	0.431	0.941	0.048	0.162	0.456	0.694	0.055
Chronic neuromuscular disease	151 (2.0)	61 (2.5)	33 (2.3)	11 (4.7)	7 (3.9)	1 (2.2)	9 (1.7)	1 (3.0)
*P* vs. influenza negative	–	0.138	0.451	0.005	0.079	0.603	0.599	0.481
Active neoplasm	245 (3.3)	59 (2.4)	33 (2.3)	12 (5.1)	3 (1.7)	0 (0.0)	11 (2.1)	0 (0.0)
*P* vs. influenza negative	–	0.038	0.061	0.125	0.289	0.404	0.124	0.626
Chronic liver disease	103 (1.4)	34 (1.4)	28 (2.0)	3 (1.3)	1 (0.6)	1 (2.2)	1 (0.2)	0 (0.0)
*P* vs. influenza negative	–	0.917	0.086	1.0	0.522	0.467	0.015	1.0
Autoimmune disease	75 (1.0)	29 (1.2)	19 (1.3)	6 (2.6)	1 (0.6)	0 (0.0)	3 (0.6)	0 (0.0)
*P* vs. influenza negative	–	0.411	0.253	0.022	1.0	1.0	0.489	1.0
Pregnant (women 15–45 y)	337 (4.5)	278 (11.5)	188 (13.3)	22 (9.4)	2 (1.1)	0 (0.0)	62 (11.7)	4 (12.1)
*P* vs. influenza negative	–	< 0.001	< 0.001	0.014	0.107	0.062	< 0.001	0.373
Obese^b,c^	1089 (14.6)	336 (13.9)	208 (14.7)	25 (10.6)	31 (17.2)	11 (24.4)	59 (11.1)	4 (12.1)
*P* vs. influenza negative	–	0.47	0.84	0.124	0.311	0.063	0.025	1.0
Outpatient consultations within ≤3 mo^d^								
0	1478 (19.8)	673 (27.9)	447 (31.6)	44 (18.7)	56 (31.1)	8 (17.8)	120 (22.6)	7 (21.2)
1	1305 (17.5)	468 (19.4)	303 (21.4)	27 (11.5)	43 (23.9)	7 (15.6)	86 (16.2)	4 (12.1)
≥ 2	2880 (38.6)	920 (38.1)	556 (39.3)	82 (34.9)	81 (45.0)	18 (40.0)	171 (32.1)	22 (66.7)
*P* vs. influenza negative	–	< 0.001	< 0.001	0.284	0.207	0.914	0.039	0.238
Smoking (≥ 18 y)^e^								
Never smoked	1881 (25.2)	667 (27.6)	391 (27.6)	96 (40.9)	35 (19.4)	17 (37.8)	121 (22.7)	9 (27.3)
Past smoker	1104 (14.8)	300 (12.4)	180 (12.7)	41 (17.4)	14 (7.8)	5 (11.1)	60 (11.3)	5 (15.2)
Current smoker	897 (12.0)	241 (10.0)	141 (10.0)	14 (6.0)	24 (13.3)	5 (11.1)	55 (10.3)	4 (12.1)
*P* vs. influenza negative	–	< 0.001	0.006	< 0.001	0.111	0.371	0.582	1.0
Functional impairment status (Barthel index, ≥ 65 y)^f^								
Total (0–15)	160 (2.1)	20 (0.8)	7 (0.5)	5 (2.1)	4 (2.2)	1 (2.2)	4 (0.8)	0 (0.0)
Severe (20–35)	69 (0.9)	8 (0.3)	2 (0.1)	2 (0.9)	0 (0.0)	1 (2.2)	3 (0.6)	0 (0.0)
Moderate (40–55)	129 (1.7)	25 (1.0)	14 (1.0)	6 (2.6)	2 (1.1)	0 (0.0)	4 (0.8)	0 (0.0)
Mild (60–90)	469 (6.3)	85 (3.5)	35 (2.5)	25 (10.6)	10 (5.6)	0 (0.0)	13 (2.4)	3 (9.1)
Minimal (95–100)	847 (11.3)	201 (8.3)	107 (7.6)	24 (10.2)	11 (6.1)	5 (11.1)	52 (9.8)	4 (12.1)
*P* vs. influenza negative	–	0.021	0.002	0.244	0.55	0.17	0.053	0.893
Time from onset of symptoms to swabbing^g^								
0–2 d	2384 (31.9)	1017 (42.1)	663 (46.9)	89 (37.9)	68 (37.8)	10 (22.2)	188 (35.3)	11 (33.3)
3–4 d	2514 (33.7)	797 (33.0)	446 (31.5)	68 (28.9)	70 (38.9)	15 (33.3)	189 (35.5)	15 (45.5)
5–7 d	2260 (30.3)	543 (22.5)	277 (19.6)	68 (28.9)	33 (18.3)	20 (44.4)	144 (27.1)	5 (15.2)
8–9 d	300 (4.0)	50 (2.1)	24 (1.7)	8 (3.4)	7 (3.9)	0 (0.0)	11 (2.1)	1 (3.0)
*P* vs. influenza negative	–	< 0.001	< 0.001	0.222	0.009	0.107	0.031	0.229
Influenza vaccination ≥14 d from ILI onset^h,i^	1235 (16.5)	243 (10.1)	108 (7.6)	22 (9.4)	17 (9.4)	6 (13.3)	81 (15.2)	11 (33.3)
*P* vs. influenza negative	–	0.207	0.559	0.007	0.41	1.0	1.0	1.0

*Abbreviations*: *ILI* influenza-like illness, *IQR* interquartile range

^a^1 missing for influenza negative

^b^Determined from the body mass index according to age and sex following the World Health Organization guidelines [12]

^c^12 missing for influenza negative, 10 for A(H1N1)pdm09 and 7 for A(H3N2)

^d^1 missing for any influenza and 1 for A(H1N1)pdm09; Beijing not included

^e^11 missing for influenza negative, 17 for any influenza, 10 for A(H1N1)pdm09, and 7 for A(H3N2)

^f^222 missing for influenza negative, 35 for any influenza, 11 for A(H1N1)pdm09, 14 for A(H3N2), 1 for B/Yamagata, and 9 for B/Victoria

^g^9 missing or > 10 d for influenza negative, 8 for any influenza, 5 for A(H1N1)pdm09, 2 for A(H3N2), 2 for A not subtyped, and 1 for B not subtyped

^h^Only vaccinated patients

^i^2 missing for any influenza, 1 for A(H3N2), and 1 for B/Victoria

**Fig. 3:**
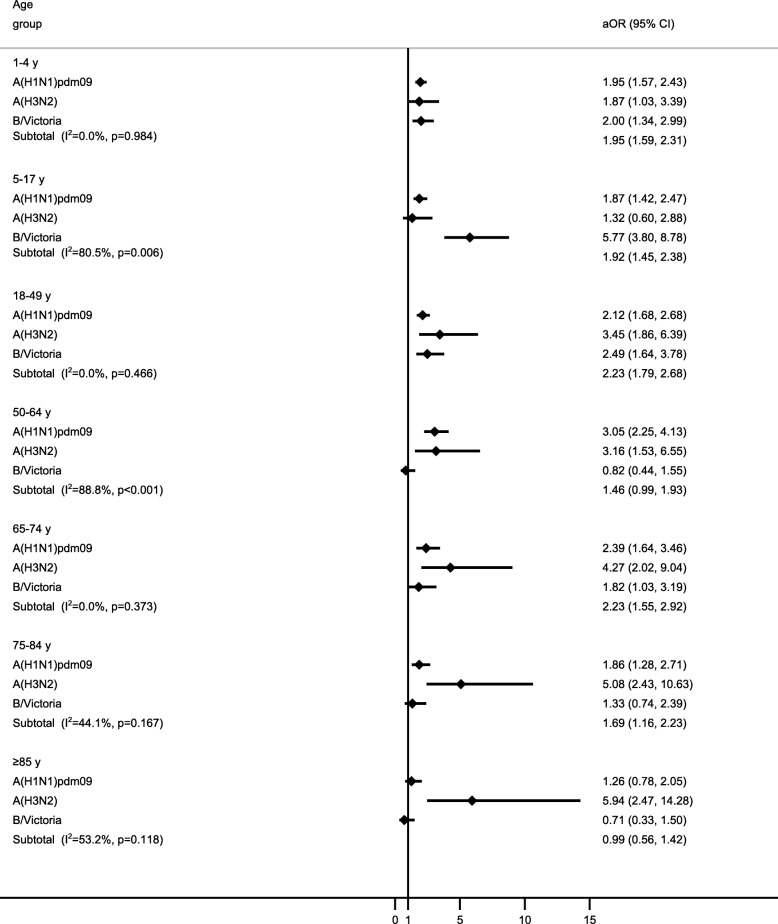
Adjusted odds ratio (aOR) by age group and strain. Adjusted by age, sex, socioeconomic class, number of chronic conditions, vaccination status, time from onset of symptoms to swabbing, and site. Abbreviation: CI, confidence interval

Heterogeneity due to strain was assessed considering A(H1N1)pdm09, A(H3N2), and B/Victoria-lineage using the I^2^ statistic with adjustment for sex, social class according to occupation, comorbidity, influenza vaccination, time to swab, and site. By age, I^2^ was 0.0% for admissions 1–4, 18–49, and 65–74 years, 80.5% for 5–17 years, 88.8% for 50–64 years; 44.1% for 75–84 years; and 53.2% for ≥85 years (Fig. 3).

### Admission with influenza according to sex and variability by influenza virus

In general, the sex distribution differed significantly between influenza-positive and influenza-negative admissions (Table 3). The risk of admission with influenza was not heterogeneous by strain after adjusting by age, social class according to occupation, comorbidity, influenza vaccination, time to swab, and site, there was no heterogeneity by strain (I^2^ = 0.0%; data not shown). This was also found when pregnant women were excluded (I^2^ = 0.0%; data not shown).

### Admission with influenza according to presence of comorbidity

Older adults positive for influenza were more likely to have underlying chronic conditions (Fig. 2). However, chronic conditions were reported significantly less frequently for influenza-positive (28.1%) than influenza-negative admissions (34.8%) (*P* <  0.001; Table 3). This was also found after excluding pregnant women (29.6% for influenza-positive vs. 35.9% for influenza-negative; *P* <  0.001) (data not shown).

**Fig. 4:**
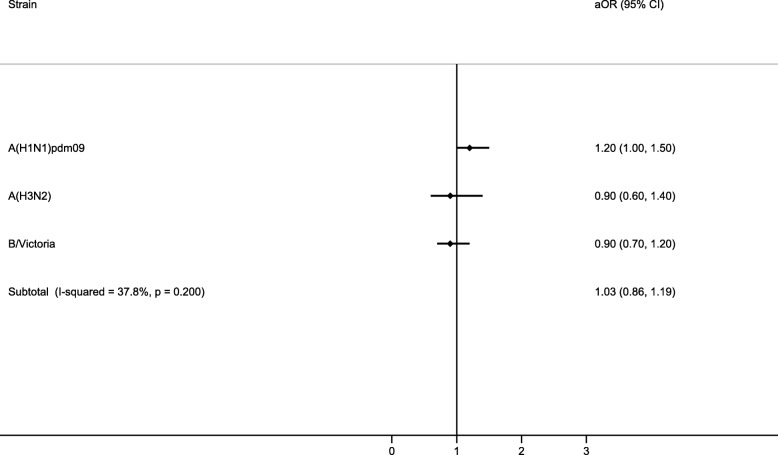
Adjusted odds ratio (aOR) by strain in admissions with underlying conditions. Adjusted by age, sex, socioeconomic class, obesity status, vaccination status, time from onset of symptoms to swabbing, and site. Abbreviation: CI, confidence interval

The adjusted OR for admission with influenza was 1.03 (95% confidence interval [CI], 0.86 to 1.19) for patients with comorbidities. No significant heterogeneity by strain was detected (I^2^ = 37.8%) (Fig. 4).

### Admission with influenza according to pregnancy

A total of 1051 women 15–45 years old were included in the study, of whom 615 were pregnant (596 in Moscow, 2 in the Czech Republic, 2 in Turkey, 4 in France, 3 in Valencia, 4 in India, and 4 in Curitiba; Table 2) and 436 were not (156 in St. Petersburg, 5 in Moscow, 25 in the Czech Republic, 20 in Turkey, 15 in France, 95 in Beijing, 69 in Valencia, 17 in Mexico, 31 in India, and 3 in Curitiba; data not shown). The probability of laboratory-confirmed influenza was significantly higher (*P* <  0.001) in included pregnant women (45.2%) than non-pregnant women in this age range (23.8%) (data not shown).

After considering site as a random effect and excluding data from St. Petersburg, Beijing, and Mexico (where no pregnant women were enrolled), the crude OR of admission with influenza was 2.82 (95% CI, 1.90 to 4.19) for pregnant women (data not shown). When considering pregnant women with no comorbidities, the crude OR was 2.64 (95% CI, 1.55 to 4.47). For pregnant women with comorbidities, the crude OR was 3.84 (95% CI, 1.96 to 7.53). There was no evidence of an interaction between comorbidity and pregnancy (*P* = 0.39), but there was evidence of confounding (*P* <  0.001).

**Fig. 5:**
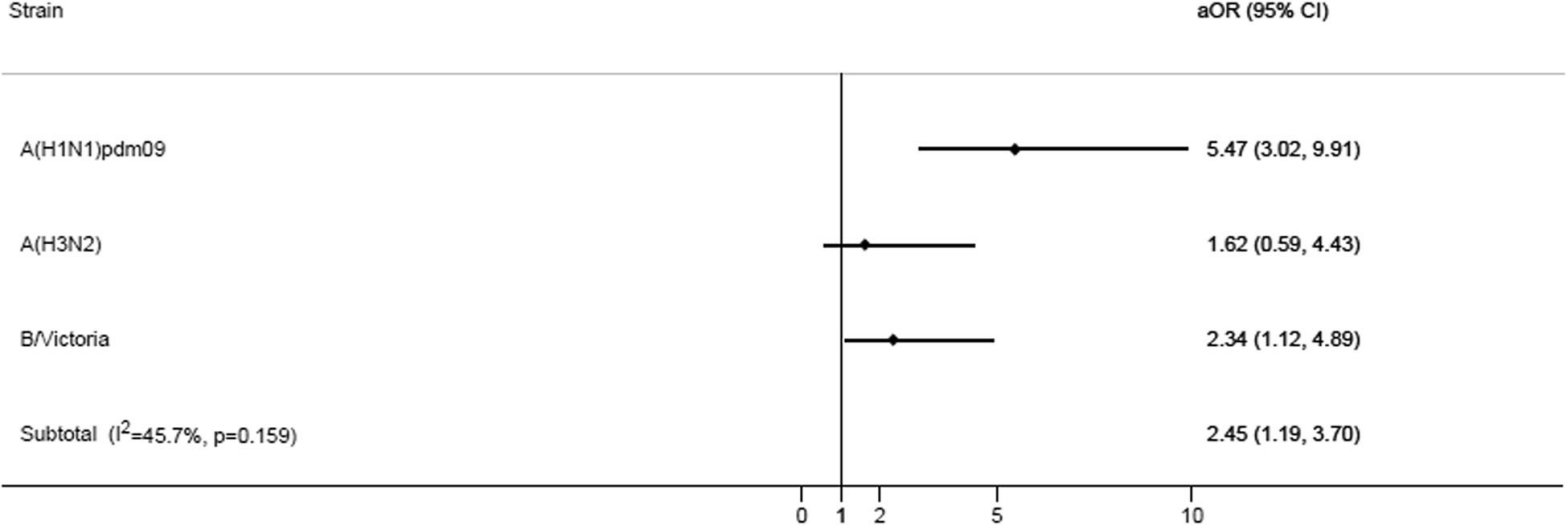
Adjusted odds ratio (aOR) by strain in pregnant admissions 15 to 45 years of age. Adjusted by presence of comorbidities. Only in women aged 15–45 years. Abbreviation: CI, confidence interval

The heterogeneity among strains detected in admitted pregnant women was low to moderate (I^2^ = 45.7%). This was due to a higher adjusted OR for admission with A(H1N1)pdm09 when adjusted for the presence of comorbidities (Fig. 5).

### Patients included in the IVE analysis

Patients with vaccine contraindications (egg allergy or < 6 months of age) or previous laboratory-confirmed influenza in the same season were excluded from the IVE analysis. After applying these exclusions, 8971 samples obtained from patients hospitalized from December, 2015 to May, 2016 were included. Of these, 2269 (25.3%) were positive for influenza. By strain, this included 1327 (58.5%) positive for A(H1N1)pdm09, 511 (22.5%) for B/Victoria-lineage, 224 (9.9%) for A(H3N2), and 41 (1.81%) for B/Yamagata-lineage (Table 4). Overall, 10.8% (*n* = 246) of influenza-positive admissions and 18.7% (*n* = 1250) of influenza-negative admissions were vaccinated (*P* <  0.001) (Table 5).

**Table 4:** IVE by age and strain overall and for admissions targeted for influenza vaccination

Population	Strain	Age	Influenza-positive		Influenza-negative		Crude IVE		Adjusted IVE^a^	
			Total	Vaccinated	Total	Vaccinated	Percent (95% CI)	P interaction	Percent (95% CI)	P interaction
Overall	Any	Any	2269	246	6702	1250	47.0 (38.6, 54.2)		16.3 (0.4, 29.7)	
		< 65 y	1897	100	4814	354	29.9 (11.9, 44.2)	0.90	11.9 (−12.9, 31.3)	0.70
		≥ 65 y	372	146	1888	896	28.5 (10.2, 43.0)		13.4 (−12.7, 33.5)	
	A(H1N1)pdm09	Any	1327	110	6702	1250	60.6 (51.6, 67.9)		36.0 (18.0, 50.1)	
		< 65 y	1153	37	4814	354	58.2 (41.0, 70.4)	0.0054	44.8 (19.2, 62.3)	0.69
		≥ 65 y	174	73	1888	896	20.0 (−9.6, 41.6)		5.7 (−36.4, 34.8)	
	A(H3N2)	Any	224	22	6702	1250	52.5 (25.9, 69.6)		16.1 (− 35.9, 48.2)	
		< 65 y	148	6	4814	354	46.8 (−21.4, 76.7)	0.26	40.2 (−39.5, 74.4)	0.44
		≥ 65 y	76	16	1888	896	70.5 (48.2, 83.2)		6.8 (−73.3, 49.9)	
	A not subtyped	Any	153	16	6702	1250	49.1 (14.2, 69.8)		49.5 (5.5, 73.0)	
		< 65 y	128	8	4814	354	16.0 (− 73.2, 59.3)	0.40	−3.7 (− 127.9, 52.8)	0.12
		≥ 65 y	25	8	1888	896	47.9 (−21.4, 77.6)		67.6 (19.4, 87.0)	
	B/Yamagata	Any	41	6	6702	1250	25.2 (−78.2, 68.6)		−96.9 (− 406.0, 23.4)	
		< 65 y	33	4	4814	354	−73.8 (− 397.2, 39.3)	0.11	–	0.59
		≥ 65 y	8	2	1888	896	63.1 (−83.5, 92.6)		–	
	B/Victoria	Any	511	83	6702	1250	15.4 (−7.9, 33.7)		−49.3 (−99.5, −11.7)	
		< 65 y	426	39	4814	354	−27.0 (−79.6, 10.2)	0.82	−32.9 (−94.0, 9.0)	0.29
		≥ 65 y	85	44	1888	896	−18.8 (−83.6, 23.1)		−1.8 (−67.2, 38.0)	
	B not subtyped	Any	29	11	6702	1250	− 166.5 (− 466.0, −25.5)		−322.2 (− 948.5, −70.0)	
		< 65 y	22	7	4814	354	− 488.0 (− 1354.1, − 137.7)	0.14	–	0.65
		≥ 65 y	7	4	1888	896	−47.6 (− 561.7, 67.1)		–	
Individuals targeted for influenza vaccination	Any	Any	1106	189	3554	1065	51.8 (42.7, 59.5)		16.2 (−3.6, 32.2)	
		< 65 y	734	43	1666	169	44.9 (22.0, 61.1)	0.21	5.3 (−39.4, 35.6)	0.30
		≥ 65 y	372	146	1888	896	28.5 (10.2, 43.0)		13.4 (−12.7, 33.5)	
	A(H1N1)pdm09	Any	646	93	3554	1065	60.7 (50.4, 68.9)		23.0 (−3.3, 42.6)	
		< 65 y	472	20	1666	169	60.8 (36.8, 75.7)	0.011	20.9 (−36.0, 54.0)	0.54
		≥ 65 y	174	73	1888	896	20.0 (−9.6, 41.6)		5.7 (−36.4, 34.8)	
	A(H3N2)	Any	137	19	3554	1065	62.4 (38.5, 77.0)		−9.1 (−88.4, 36.8)	
		< 65 y	61	3	1666	169	54.2 (−48.0, 85.8)	0.52	−2.5 (− 263.0, 71.1)	0.10
		≥ 65 y	76	16	1888	896	70.5 (48.2, 83.2)		6.8 (−73.3, 49.9)	
	A not subtyped	Any	71	12	3554	1065	52.5 (11.2, 74.6)		61.7 (21.6, 81.3)	
		< 65 y	46	4	1666	169	15.6 (−138.2, 70.1)	0.49	25.7 (− 129.6, 76.0)	0.50
		≥ 65 y	25	8	1888	896	47.9 (−21.4, 77.6)		67.6 (19.4, 87.0)	
	B/Yamagata	Any	26	5	3554	1065	44.4 (−48.0, 79.1)		−104.3 (− 508.0, 31.4)	
		< 65 y	18	3	1666	169	−77.2 (− 518.6, 49.3)	0.13	− 115.4 (− 747.1, 45.2)	0.94
		≥ 65 y	8	2	1888	896	63.1 (−83.5, 92.6)		–	
	B/Victoria	Any	215	55	3554	1065	19.7 (−10.1, 41.4)		−25.6 (−86.3, 15.4)	
		< 65 y	130	11	1666	169	18.1 (−55.0, 56.7)	0.33	−17.3 (−144.8, 43.8)	0.71
		≥ 65 y	85	44	1888	896	−18.8 (− 83.6, 23.1)		−1.8 (−67.2, 38.0)	
	B not subtyped	Any	17	7	3554	1065	−63.6 (−331.1, 37.9)		–	
		< 65 y	10	3	1666	169	− 279.6 (− 1385.0, 3.0)	0.37	–	–
		≥ 65 y	7	4	1888	896	−47.6 (−561.7, 67.1)		–	

*Abbreviations*: *CI* 95% CI, *IVE* influenza vaccine effectiveness

^a^Adjust for age, sex, number of chronic conditions, time from onset of symptoms to swab, epidemiological week at admission, and site

**Table 5:** Characteristics of patients included in the IVE analysis by vaccination status

Risk variable	Category	Unvaccinated	Vaccinated	*P* value
Number of patients, n (%)	Controls	5452 (72.9)	1250 (83.6)	< 0.001
	Cases	2023 (27.1)	246 (16.4)	
Age (y)	Median (IQR)	19.3 (3.0–51.9)	74.8 (56.3–83.3)	< 0.001
Age group, n (%)	6–11 months	524 (7.0)	14 (0.9)	< 0.001
	1–4 y	2370 (31.7)	76 (5.1)	
	5–17 y	758 (10.1)	136 (9.1)	
	18–49 y	1879 (25.1)	114 (7.6)	
	50–64 y	726 (9.7)	114 (7.6)	
	65–74 y	487 (6.5)	302 (20.2)	
	75–84 y	513 (6.9)	438 (29.3)	
	≥ 85 y	218 (2.9)	302 (20.2)	
Female, n (%)	–	3480 (46.6)	637 (42.6)	0.005
Comorbidities, n (%)	No	5350 (71.6)	415 (27.7)	< 0.001
	Yes	2125 (28.4)	1081 (72.3)	
Pregnant (women 15–45 y), n (%)	–	606 (59.4)	8 (29.6)	0.002
Obese^a^, n (%)	–	1038 (13.9)	321 (21.5)	< 0.001
Hospitalization within ≤12 mo, n (%)	–	1954 (26.1)	549 (36.7)	< 0.001
Outpatient consultations within ≤3 mo, n (%)	No	2624 (35.11)	304 (20.32)	< 0.001
	Yes	4850 (64.89)	1192 (79.68)	
Smoking (≥ 18 y), n (%)	Current	949 (25.0)	186 (14.7)	< 0.001
	Past	924 (24.3)	475 (37.5)	
	Never	1926 (50.7)	605 (47.8)	
Functional impairment (≥ 65 y), n (%)	None or minimal	471 (46.0)	572 (58.3)	< 0.001
	Mild	321 (31.4)	232 (23.7)	
	Moderate	98 (9.6)	54 (5.5)	
	Severe	38 (3.7)	39 (4.0)	
	Total	95 (9.3)	84 (8.6)	
Sampling interval (d)	Median (IQR)	3 (2–5)	4 (2–5)	< 0.001
Sampling interval, n (%)	≤ 4 d	5139 (68.8)	920 (61.5)	
	5–7 d	2104 (28.2)	475 (31.8)	
	8–9 d	232 (3.1)	101 (6.8)	
Site, n (%)	St. Petersburg	1645 (22.0)	45 (3.0)	< 0.001
	Moscow	1808 (24.2)	87 (5.8)	
	Czech Republic	115 (1.5)	7 (0.5)	
	France	81 (1.1)	60 (4.0)	
	Turkey	522 (7.0)	43 (2.9)	
	Beijing	1854 (24.8)	258 (17.3)	
	Valencia	1018 (13.6)	931 (62.2)	
	India	259 (3.5)	15 (1.0)	
	Mexico	87 (1.2)	5 (0.3)	
	Curitiba	86 (1.2)	45 (3.0)	
Vaccinated, n (%)	In 2013–2014	526 (7.1)	1073 (72.8)	< 0.001
	In 2014–2015	545 (7.4)	1200 (81.2)	< 0.001

^a^Determined from the body mass index according to age and sex following the World Health Organization guidelines [12]

The proportion of patients vaccinated with the seasonal influenza vaccine ≥14 days before symptom onset was 2.7% (*n* = 45) in St. Petersburg, 4.6% (*n* = 87) in Moscow, 5.7% (*n* = 7) in the Czech Republic, 7.6% (*n* = 43) in Turkey, 42.6% (*n* = 60) in France, 12.2% (*n* = 258) in Beijing, 47.8% (*n* = 931) in Valencia, 5.4% (*n* = 5) in Mexico, 5.5% (*n* = 15) in India, and 34.4% (n = 45) in Curitiba (data not shown).

Vaccinated admissions were older (median age = 74.8 years) than unvaccinated admissions (median age = 19.3 years) (*P* <  0.001) (Table 5). The proportion of participants with underlying conditions was significantly higher in vaccinated admissions (72.3%) than in unvaccinated admissions (28.4%) (*P* < 0.001). Also, the proportion of individuals considered obese was higher for vaccinated (21.5%) than for unvaccinated (13.9%) admissions (*P* < 0.001). Vaccination was more common in individuals hospitalized in the previous 12 months than in those who had not been (*P* < 0.001) and more common in individuals who visited a general practitioner within the last 3 months than those who did not (*P* < 0.001). Only 8 of the 614 pregnant patients (1.3%) were vaccinated. Most patients vaccinated in the 2015–2016 influenza season reported prior influenza vaccination: 72.8% were also vaccinated in 2013–2014, and 81.2% were also vaccinated in 2014–2015.

### IVE in overall admissions and in patients targeted for influenza vaccination

Against all-age influenza-related hospitalization, the crude IVE was 47.0% (95% CI, 38.6 to 54.2%) overall (Table 4). After adjusting for age, sex, number of chronic conditions, time from onset of symptoms to swabbing, epidemiological week at admission, and site, IVE was 16.3% (95% CI, 0.4 to 29.7%) overall. By strain, the adjusted IVE was 36.0% (95% CI, 18.0 to 50.1%) against A(H1N1)pdm09, 16.1% (95% CI, − 35.9 to 48.2%) against A(H3N2) and − 49.3% (95% CI,− 99.5 to − 11.7%) against the B/Victoria-lineage. Considering these three predominant strains, IVE heterogeneity between strains was substantial (I^2^ for adjusted IVE = 84.6%; data not shown). Reliable IVE estimates could not be made by age group for B/Yamagata or B not subtyped because these strains were not frequently detected (Table 4). IVE differed little between younger and older patients in the overall population: the adjusted IVE was 11.9% (95% CI, − 12.9 to 31.3%) for patients < 65 years of age and 13.4% (95% CI, − 12.7 to 33.5%) for patients ≥65 years of age (Table 4). Differences in adjusted IVE between the two age groups for individual strains were not statistically significant.

Results were similar when restricting the analysis to patients targeted for influenza vaccination: the crude IVE for all ages was 51.8% (95% CI, 42.7 to 59.5%) and the adjusted IVE was 16.2% (95% CI, − 3.6 to 32.2%) (Table 4). By strain, the adjusted IVE was 23.0% (95% CI, − 3.3 to 42.6%) against A(H1N1)pdm09, − 9.1% (95% CI, − 88.4 to 36.8%) against A(H3N2), and − 25.6% (95% CI, − 86.3 to 15.4%) against B/Victoria lineage.

### IVE across sites

**Fig. 6:**
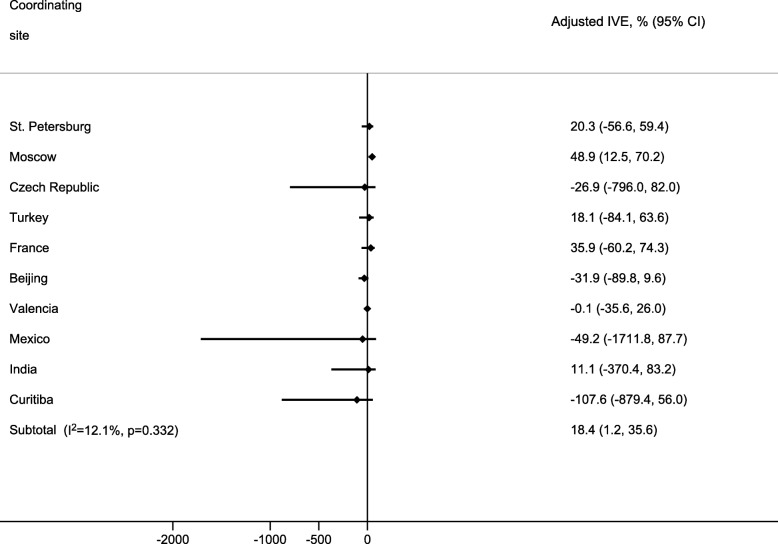
Adjusted influenza vaccine effectiveness (IVE) by site. Adjusted by age, sex, number of chronic conditions, time from onset of symptoms to swabbing and epidemiological week at admission. Abbreviation: CI, confidence interval

The highest overall IVE among all hospitalizations was detected in Moscow, followed by France, St. Petersburg, Turkey, and India (Fig. 6). The lowest IVE was detected in Curitiba, followed by Mexico, Beijing, Czech Republic, and Valencia. IVE was only significant in Moscow. Between sites, heterogeneity in the estimates of IVE against influenza-related hospitalization was low (I^2^ for adjusted IVE = 12.1%).

The influenza vaccines available and target populations in each represented country are provided in Additional file 3: Table S3.

## Discussion

Data collected by active surveillance within the GIHSN sites indicated that during the 2015–2016 influenza season (week 48 of 2015 to week 33 of 2016), the predominant circulating strain in hospitalized individuals was A(H1N1)pdm09 followed by B/Victoria-lineage and A(H3N2) strains. B/Yamagata-lineage strains were relatively rare. This agrees with overall patterns of influenza circulation reported by the World Health Organization and others [13–17]. The predominance of influenza A(H1N1)pdm09 in Saint Petersburg (Russia), Moscow (Russia), Czech Republic, Turkey, France, Valencia (Spain), Mexico and Curitiba (Brazil), and B/Victoria-lineage in Beijing (China), agree with data reported for these countries [18–24]. Regional activity for A(H3N2) was reported in India, coinciding with our data obtained in the country’s Jammu and Kashmir state [24].

During the 2015–2016 season, admissions with laboratory-confirmed influenza were younger than those who were negative for influenza, and about three-quarters (76%) of influenza-positive admissions were < 50 years of age. As found by the GIHSN in 2014–2015 season [8], among patients with laboratory-confirmed influenza, those positive for A(H1N1)pdm09 were younger than those positive for A(H3N2) or B/Yamagata-lineage, although patients positive for B/Victoria-lineage were the youngest. Patients with underlying conditions were more prone to be infected by A(H1N1)pdm09 or A(H3N2) than by B/Victoria-lineage. However, most (72%) of the influenza-positive patients did not have chronic conditions (Fig. 2). These results confirm that healthy and young individuals are susceptible to severe influenza, in agreement with our previous reports [5–8].

As reported previously in the GIHSN [7, 8] and in a recent meta-analysis [25], hospitalized women were more likely to be positive for influenza if they were pregnant. However, only 8 out of 614 (1.3%) pregnant women were vaccinated against influenza. The vaccination coverage rate was also low for the overall study population (15.4%).

According to the current study, IVE in 2015–2016 was low to moderate against hospitalization with laboratory-confirmed influenza (adjusted IVE = 16.3% [95% CI, 0.4 to 29.7%]) and did not differ significantly between younger and older patients. By comparison, the GIHSN reported overall adjusted IVEs against hospitalization with laboratory-confirmed influenza of 33% (95% CI, 11 to 49%) in 2012–2013 [5] and 22% (95% CI, 8 to 33%) in 2014–2015 [8]. Other reports from Hong Kong, the US, and Finland have shown a higher IVE for the 2015–2016 season (IVEs ~ 50%–70%) than that reported in the current study [26–29]. We speculate that the differences in IVE estimates are related to a combination of different circumstances; among them, the variable genetic and antigenic characteristics of the A(H1N1)pdm09 and A(H3N2) emerging clades circulating in different parts of the world [30–32], the potential inadequacy of the response to egg-derived vaccines [33], the mismatch between the vaccine and circulating B viruses [34], and the age composition compounded with low levels of vaccination in our population [35].

The highest IVE was against A(H1N1)pdm09 (adjusted IVE = 36.0% [95% CI, 18.0 to 50.1%), in agreement with reports that the circulating A(H1N1)pdm09 virus was antigenically similar to the vaccine strain [15]. Despite a new emerging A(H1N1)pdm09 6B.1 subclade detected in Spain, Canada, and Denmark [30, 31, 36], several other studies have similarly reported moderate effectiveness against A(H1N1)pdm09 in different age groups and healthcare settings [26, 28, 31, 36–38].

We were unable to obtain reliable IVE estimates for B/Yamagata-lineage or B not subtyped by age group because these strains were not frequently detected. The vaccine had no effect on illness caused by B/Victoria-lineage viruses, which could be due to the absence of a B/Victoria-lineage strain from the WHO-recommended trivalent inactivated vaccine for the northern hemisphere 2015–2016 season [39]. This also suggests that any cross-lineage antibody response from the trivalent vaccine B/Yamagata-lineage strain was insufficient to protect against illness caused by circulating B/Victoria-lineage strains. Although we obtained one isolated negative IVE in preventing admissions with B/Victoria-lineage, all other IVE estimates for the B/Victoria-lineage consistently showed no effect, and so emphasizing this isolated negative IVE would incorrectly reject the null hypothesis [40]. A multi-comparison adjustment could have been used to solve this, though we choose to follow the reasoning of other authors that endorse the reporting of all results, not adjusting for multiple comparisons [41, 42]. These findings are consistent with several other reports for the 2015–2016 season [27, 36, 43] and highlight the need for quadrivalent vaccines containing both B lineages [44–46].

We observed substantial differences between crude and adjusted IVE estimates. This is likely because young adults have a lower probability of vaccination and underlying conditions compared to older subjects, whereas older adults are more often vaccinated and experience an increased risk of adverse health effects due to age and underlying comorbidities (as shown in Fig. 2). Therefore, when no age distinction is made in the analysis (i.e., no adjustment or stratification by age) this bias leads to a higher vaccination efficacy and, accordingly, the estimate adjusted by age and stratified by age group (i.e., < 65 and ≥ 65 years) is lower than the crude (unadjusted, not stratified) estimate. Indeed, some authors consider it improper to report crude estimates at all [47].

### Limitations and considerations

As described previously [7], results from the GIHSN should be interpreted with caution due to the heterogeneity and bias of multi-centric observational studies. The GIHSN takes heterogeneity into account by using a test-negative design that compares laboratory-confirmed influenza admissions with influenza-negative admissions, and by restricting the analysis to periods with influenza circulation, adjusting modeling, and accepting only data from patients admitted within 7 days of the onset of ILI symptoms. In addition, all participating sites follow a standardized protocol that is regularly reviewed and reinforced during the GIHSN annual general meeting and on-site visits at each participating hospital. Through this common core protocol, the ILI case definition used in our study was the same between different study sites, and selection bias was minimized by enrolling consecutive admissions without knowing their vaccination status or the laboratory results for influenza infection.

In addition, different sources of data were used to ensure complete case ascertainment, including data from clinical records, health registries, and information provided by the patient and attending nurses and doctors. Influenza vaccination status was obtained by asking the patient (or representative) if they had received the current season’s influenza vaccine, the date of vaccination, and if the vaccine had been administered at least 2 weeks before the onset of symptoms. Additionally, when records existed, this information was validated by existing registers, vaccination cards, or through contacting the clinic where the vaccine was administered. To describe heterogeneity we reported the I^2^ parameter. Finally, we used random effects to account for variability by site.

From 2014-2015 to 2015–2016, the GIHSN expanded from seven coordinating sites in six countries to 11 coordinating sites in nine countries. Even though the GIHSN has expanded, the total number of included admissions decreased slightly from the previous year: in 2015–2016, 18,360 eligible admissions were identified of which 9882 (53.8%) met the inclusion criteria, whereas in 2014–2015, 23,551 eligible admissions of which 9614 (40.8%) met the inclusion criteria. Therefore, for some analyses, small numbers continue to be a limitation. The ability to pool data across the GIHSN sites helps, although further improvement will depend on the continued expansion of the network and the growing experience of the participating sites.

## Conclusions

The 2015–2016 influenza season was dominated by A(H1N1)pdm09, followed by B/Victoria-lineage and A(H3N2), with few cases of B/Yamagata-lineage. Many of the influenza hospitalizations were for young, otherwise healthy individuals, and as in previous years, hospitalized women were more likely to be positive for influenza if they were pregnant. During the 2015–2016 season, influenza vaccines provided low to moderate protection against hospitalization with influenza and no protection against the predominant circulating B lineage, highlighting the need for more effective and broader influenza vaccines.

## Additional files

Additional file 1: **Table S1.** Signs and symptoms required for enrollment in patients less than five years of age. (DOCX 17 kb)

Additional file 2: **Table S2.** Time periods of patient enrolment for each study site. (DOCX 17 kb)

Additional file 3: **Table S3.** Vaccines available and targeted groups for vaccination in the GIHSN participating sites. (DOC 48 kb)

## Abbreviations

CI: Confidence interval
GIHSN: Global Influenza Hospital Surveillance Network
ILI: Influenza-like illness
IVE: Influenza vaccine effectiveness
OR: Odds ratio
