# Supplementary material for: Influenza epidemiology and influenza vaccine effectiveness during the 2015–2016 season: results from the Global Influenza Hospital Surveillance Network
Source: BMC Infect Dis. 2019 May 14;19:415. doi: 10.1186/s12879-019-4017-0 (PMC6518734; doi:10.1186/s12879-019-4017-0)
Supplement: Supplementary file 1 — Table S1. Signs and symptoms required for enrollment in patients less than five years of age. (DOCX 17 kb) [file 12879_2019_4017_MOESM1_ESM.docx]

**Table S1**. Signs and symptoms required for enrollment in patients less than 5 years of age

| **Sign or symptom** | **ICD 9 Codes** | **ICD 10 Codes** |
| --- | --- | --- |
| Acute upper or lower respiratory disease | 382.9; 460 to 466 | J00-J06, J20-J22 |
| Dyspnea, breathing anomaly, shortness of breath, tachypnea (polypnea) | 786.0; 786.00; 786.05-786.07; 786.09; 786.9 | R06.0, R06, R06.9, R06.3, R06.00, R06.09, R06.83, R06.02, R06.82, R06.2, R06.89 |
| Acute asthma or exacerbation | 493.92 | J45.901 |
| Pneumonia and influenza | 480 to 488 | J09-J18 |
| Acute respiratory failure | 518.82 | J96 |
| Acute heart failure | 428-429.0 | I50-I50.9; I51.4 |
| Myalgia | 729.1 | M79.1 |
| Altered consciousness, convulsions, febrile convulsions | 780.01-780.02; 780.09; 780.31-780.32 | R40.20, R40.4, R40.0, R40.1, R56.00, R56.01 |
| Fever or fever unknown origin or non specified | 780.6-780.60 | R50, R50.9 |
| Cough | 786.2 | R05 |
| Gastrointestinal manifestations | 009.0; 009.3 | A09.0; A09.9 |
| Sepsis, Systemic inflammatory response syndrome, not otherwise specified | 995.90-995.94 | R65.10, R65.11, R65.20, A41.9 |
| Nausea and vomiting | 078.82; 787.0; 787.01-787.03 | R11; R11.0; R11.10 - R11.12; R11.2 |
