# Supplementary material for: Influenza epidemiology and influenza vaccine effectiveness during the 2015–2016 season: results from the Global Influenza Hospital Surveillance Network
Source: BMC Infect Dis. 2019 May 14;19:415. doi: 10.1186/s12879-019-4017-0 (PMC6518734; doi:10.1186/s12879-019-4017-0)
Supplement: Supplementary file 2 — Table S2. Time periods of patient enrolment for each study site. (DOCX 17 kb) [file 12879_2019_4017_MOESM2_ESM.docx]

**Table S2**. Time periods of patient enrolment for each study site

| **Site** | **Start of enrolment** | **End of enrolment** |
| --- | --- | --- |
| St. Petersburg, Russia | The week when ≥5 new influenza cases registered | The week when no laboratory-confirmed influenza cases detected |
| Moscow, Russia | Start of 2 consecutive weeks with ≥1 confirmed influenza cases | End of 2 consecutive weeks with ≤2 confirmed influenza cases |
| Czech Republic | Start of 2 consecutive weeks with ≥1 confirmed influenza cases | End of 4 consecutive weeks with no confirmed influenza cases |
| Valencia, Spain | Start of 2 consecutive weeks with ≥2 confirmed influenza cases | End of 2 consecutive weeks with no confirmed influenza cases |
| India | December, 2015 | April, 2016 |
| Mexico | October, 2015 | May, 2016 |
| Curitiba, Brazil | April, 2016 | September, 2016 |
| France | Start and end according to the Institute for Public Health Surveillance and the ‘Weekly newsletter of the Sentinels’ network’ | |
| Turkey | Start and end according to the Turkish Ministry of Health | |
| Beijing, China | During the period where influenza cases were recorded for two or more consecutive weeks | |
