# Supplementary material for: Influenza epidemiology and influenza vaccine effectiveness during the 2015–2016 season: results from the Global Influenza Hospital Surveillance Network
Source: BMC Infect Dis. 2019 May 14;19:415. doi: 10.1186/s12879-019-4017-0 (PMC6518734; doi:10.1186/s12879-019-4017-0)
Supplement: Supplementary file 3 — Table S3. Vaccines available and targeted groups for vaccination in the GIHSN participating sites. (DOC 48 kb) [file 12879_2019_4017_MOESM3_ESM.doc]

**Table S3. Vaccines available and targeted groups for vaccination in the GIHSN participating sites**

| **City (Country)** | **Names and types of vaccines 2015/16** | **Target population for influenza vaccination** |
| --- | --- | --- |
| Saint Petersburg (Russian Fed.) | “Grippol”, Russia  “Sovigripp”, Russia  Live influenza vaccine “Ultravac”, Russia  “Vaxigrip” (Sanofi Pasteur), France  “Influvac” (Abbott products), The Netherlands | Children aged ≥6 months  Students grades 1−11  Students of professional schools and high schools  Employees of medical and educational institutions, service sector, transport workers  Adults aged >60 years  Pregnant women  People with chronic cardiovascular or pulmonary conditions, metabolic disorders, and obesity |
| Moscow (Russian Fed.) | The following vaccines are recommended by National Health Authorities and used in Russia:  1. Grippol, MicroGene, Russia, polymer subunit inactivated vaccine with adjuvant polyoxidonium (recommended for all population groups aged ≥6 months)  2. Grippol plus, Petrovax, Russia, polymer subunit inactivated vaccine with adjuvant polyoxidonium (recommended for all population groups aged ≥6 months)  3. Sovigripp, MicroGene, Russia, polymer-subunit inactivated vaccine with adjuvant sovidon (recommended for all population groups aged ≥18 years)  The following vaccines are also available:  4. Influvir, MicroGene, Russia, live vaccine (from 3 years of age)  5. Grippovac, Russia, inactivated virion vaccine (from 18 years of age)  6. Vaxigrip, Sanofi Aventis, France (from 6 months of age)  7. Influvac, Abbot Biologicals, The Netherlands (from 6 months of age)  8. Fluvaxin, Changchun Changsheng Life Sciences, China (from 6 months of age) | Children from 6 months until 16 years of age (school grades 1−11)  Students of professional and vocational training institutions  Employees of medical and educational institutions, transportation, utilities  Adults aged >60 years  Pregnant women  Military personnel  Persons with chronic conditions including COPD, cardiovascular diseases, metabolic disorder, obesity, diseases and defects of the central nervous system, bronchopulmonary diseases, asthma, chronic renal disease, diabetes, autoimmune disease, allergic diseases (except allergy to chicken protein), chronic anemia, congenital or acquired immunodeficiency, HIV |
| Praha (Czech Republic) | Vaxigrip (Sanofi Pasteur)  Influvac (Abbott)  Optaflu (Novartis) | People aged ≥65 years  People of any age with chronic conditions: chronic respiratory diseases; chronic cardiovascular diseases; chronic renal diseases; chronic metabolic diseases including diabetes; chronic immunodeficiency  Vaccination also recommended to pregnant women and health care and social care workers |
| Paris/Lyon (France) | Trivalent vaccines:  Agrippal / Fluarix / Immugrip / Influvac / Vaxigrip | People aged 65 and over  Pregnancy  Obese people  People with chronic disease |
| Istanbul Bursa Ankara (Turkey) | Vaxigrip (Sanofi Pasteur) trivalent inactivated flu vaccine  Fluarix tetra (GSK) quadrivalent inactivated flu vaccine (on the market since the 2014−2015 season)  Fluarix (GSK)- trivalent inactivated flu vaccine (was on the market until 2015−2016) | In Turkey, vaccination is recommended and reimbursed for persons ≥65 years of age, people living in nursing homes and care centers for elderly, patients with chronic pulmonary diseases including asthma, patients with chronic cardiac diseases, patients with any chronic metabolic diseases including diabetes, patients with chronic renal dysfunction, adults or children with hemoglobinopathies, immune deficiency or receiving immunosuppressive treatment, adolescents and children between 6 months and 18 years who are on long-term acetylsalicylic acid treatment, and health care workers. |
| Beijing (China) | Marketed influenza vaccines in China were all trivalent inactivated influenza vaccines (TIV). There are in total 16 manufacturers in China and abroad providing seasonal influenza vaccines, which are mainly split vaccines and subunit vaccines | Children aged 6–59 months  Persons aged ≥60 years  Persons aged 5–59 years who have medical conditions that put them at higher risk for influenza-related complications  Pregnant women  Health care personnel  Household contacts and caregivers of children aged <5 years and adults aged ≥60 years  Household contacts and caregivers of persons aged 5–59 years with chronic medical conditions  Staff of kindergartens and nursery schools |
| Srinagar, Kashmir (India) | Vaxigrip  Influvac (Inactivated trivalent vaccine)  Live trivalent vaccine from Serum Institute marketed by Cipla, India (introduced this year only) | No guidelines by Ministry of Health and Family welfare recommend uniform vaccination for the population. The Ministry has recommended vaccination for Healthcare workers dealing with patients since May 2015. Other bodies like Indian academy of Pediatrics, Geriatric Association of India, and the Federation of Gynecologists and Obstetricians of India have given conditional recommendations for vaccination of individuals at high risk for influenza complications, i.e. young children, elderly adults, and pregnant women (>26 weeks) |
| Valencia (Spain) | Vaxigrip® (Sanofi Pasteur): split virus, egg based (from 6 months of age)  Chiromas® (Novartis): surface Ag, egg-based, MF59C.1 adjuvanted (for ≥65 years of age)  Optaflu® (Novartis): surface Ag, MDCK cell-based (for ≥18 years of age) | Persons aged ≥60 years, >6 months, and <60 years with underlying medical conditions. Persons who work with high risk people (e.g., in care homes, hospitals etc.), Institutionalized people, public service workers, pregnant women, children aged 6 months to 18 years on long-term aspirin treatment |
| Mexico | Fluzone and Vaxigrip (Sanofi Pasteur; inactivated trivalent vaccine)  Fluzone quadrivalent (Sanofi Pasteur; quadrivalent flu vaccine)  Fluarix (GlaxoSmithKline; trivalent flu vaccine)  Fluzactal Tetra (GlaxoSmithKline; quadrivalent flu vaccine)  Agrippal SI and Fluad (Novartis; trivalent flu vaccine)  Ollinflu (BIRMEX; trivalent flu vaccine) | According to the Mexican National Health Authorities, young children (6 months to 5 years of age) and the elderly (above 60 years of age) are priority groups. Recently, vaccination has expanded to include those aged 5 to 59 years at high risk for influenza (e.g., people with morbid obesity, diabetes, cardio-vascular disease, immunosuppressed, no spleen, hemoglobin disease, chronic renal disease, arthritis, during pregnancy, etc.) health workers, and nursery school workers. |
| Brazil (Fortaleza) | Sanofi-Pasteur Southern Hemisphere vaccines | Children between 6 months and 2 years of age, health care workers, pregnant women, indigenous people, and those with chronic diseases |
| Brazil (Curitiba) | Vaccines produced by Butantan Institute and by Sanofi Pasteur (from United States and France). | Children >6 months and <5 years age  Pregnant or puerperal women  Health Worker  Indigenous people  Individuals aged ≥60  Prisoners and penitentiary staff  Persons with chronic illnesses |
